# CT Lung Screening in Patients with Laryngeal Cancer

**DOI:** 10.1038/s41598-020-61511-3

**Published:** 2020-03-13

**Authors:** Krzysztof Piersiala, Lee M. Akst, Alexander T. Hillel, Simon R. Best

**Affiliations:** 1 Department of Otolaryngology – Head and Neck Surgery, Johns Hopkins University, Baltimore, MD USA; 2 Division of ENT Diseases, Department of Clinical Sciences, Intervention and Technology, Karolinska Institute, Stockholm, Sweden

**Keywords:** Cancer imaging, Risk factors

## Abstract

Laryngeal cancer (LC) patients who meet the age and smoking criteria of the U.S. Preventive Services Task Force (USPSTF) for annual CT lung screening were analysed for pulmonary nodules (PN) detection and secondary lung cancer (SLC) diagnosis. This is a retrospective chart review of LC patients treated at Johns Hopkins Hospital from January 2010 to December 2017. The study population included patients who met USPSTF criteria by age and smoking history for annual chest screening and were followed for at least 3 consecutive years. A total of 998 LC patients’ records were reviewed, of which 151 met the inclusion criteria. Inadequate follow-up period (37% of excluded cases) was the most common reason for exclusion, followed by not meeting USPSTF age criteria (27% excluded cases). In seventy-eight patients (n = 78, 52% of analysed patients) PN were reported. Nine individuals (6% of analysed patients) were diagnosed with SLC. Age over 70 (p = 0.003) was an independent predictor of malignancy. White race and smoking history over 40 pack-years were positively associated with a pulmonary nodule detection (p = 0.037 and p = 0.044, respectively). The incidence of PN and SLC in patients with LC is high. Many patients with laryngeal cancer meet the formal guidelines for USPSTF screening, and should be screened annually according to evidence-based medicine for the early detection of secondary lung cancers.

## Introduction

Approximately 13,150 new cases of laryngeal cancer (LC) are diagnosed every year in the USA^1^. The most pronounced risk factors remain tobacco smoking and alcohol consumption^2^, and the 5 year overall survival has not changed significantly over the last 20 years and it is currently estimated at approximately 60%^1^. One of the significant reasons for the reduced overall survival is that the incidence of secondary primary lung cancer (SPLC) in patients affected by LC ranges from 5 to 19%^3–6^, which has a significant impact on outcome. The risk of pulmonary nodules is even higher and has been reported to be up to 58%^7^ in head and neck cancer (HNC) patients.

One of the national attempts to reduce the smoking-related mortality was the introduction of the U.S. Preventive Services Task Force (USPSTF) recommendations for annual lung cancer screening with low-dose CT in a group of high-risk smokers. This screening program has proven to prevent a significant number of lung cancer–related deaths in patients who received three CT scans over the course of two years. The USPSTF recommends annual chest imaging with low-dose CT for adults aged 55–80, with at least 30 pack-years smoking history in current smokers or those who have quit within the past 15 years^8^. However, one of the exclusion criterion of the large clinical trials^9^ justifying implementation of screening program was previously known malignancy. Practically, this meant that HNC patients with substantial smoking history and obvious cancer predisposition were excluded.

The aim of this study was therefore to assess the frequency of incidental findings on CT screening such as pulmonary nodules (PN) and secondary lung cancer (SLC) in a selected group of high-risk LC patients meeting the official USPSTF criteria. We also aimed to identify the cumulative risk of PN and SLC and describe the risk factors associated with secondary lung cancer and pulmonary nodules. We hypothesized that patients with LC would have findings on lung CT at an equal frequency to smokers without LC, at rate that would justify annual lung CT screening according to evidence-based guidelines.

## Materials and Methods

### Ethics

The institutional review board of Johns Hopkins School of Medicine, Baltimore, MD, USA approved the study and accepted that informed consent was not required for this retrospective chart review. All procedures performed in studies involving human participants were in accordance with the ethical standards of the institutional and national research committee and with the 1964 Helsinki declaration and its later amendments or comparable ethical standards.

### Chart review

This is a retrospective chart review of patients diagnosed with LC who met USPSTF criteria for annual chest imaging with low dose CT and were followed for at least 3 consecutive years. All enrolled patients were treated and seen at Johns Hopkins Hospital, Baltimore, MD, USA between January 2010 and December 2017. The same database was used in the article *Clinical practice patterns in laryngeal cancer and introduction of CT lung screening*^10^.

The inclusion criteria were: being 55 to 77 years old at the time of the first recorded follow- up year, having consistent follow-up of at least 3 consecutive years in Johns Hopkins medical records system, smoking history of at least 30 pack-years in current smoker or those who quit within the past 15 years and not having synchronous lung cancer (or lugs metastases) or any other severe lung disease.

**Figure 1:**
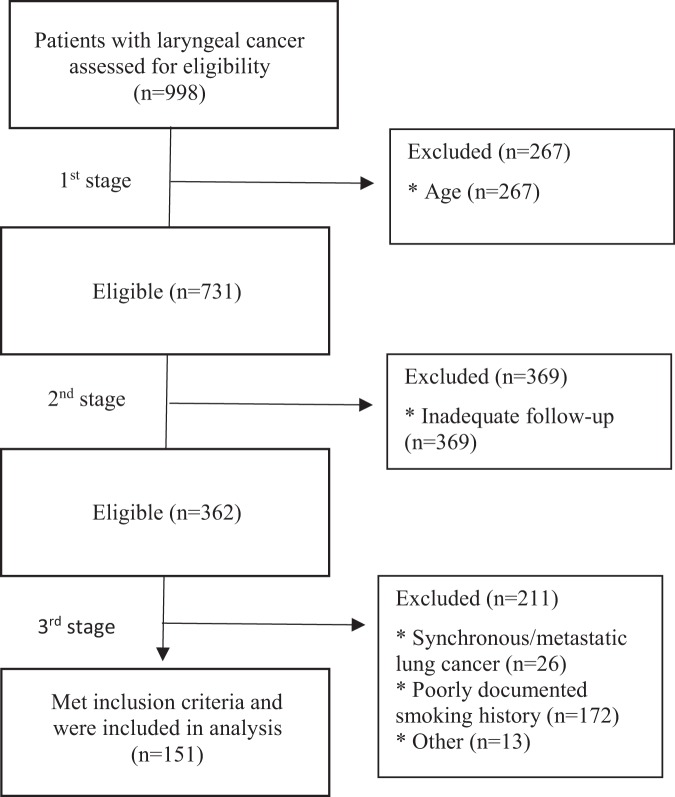
Three stage exclusion protocol to identify index patients that meet USPSTF criteria for annual chest screening. Patients were excluded in three stages: (1) age, (2) inadequate follow-up, and finally (3) smoking history or synchronous lung cancer. Of 998 initial patients, 151 fully met study criteria and were included for further analysis. *Indicates the reason for exclusion. ^#^For the purpose of the study, we defined synchronous tumors (synchronous lung cancer) as malignancies presenting within 6 months of diagnosis of the index tumors (here laryngeal cancer), and metachronous tumors (secondary lung cancer) as those presenting more than 6 months after diagnosis of the index tumor (here larygeal cancer).

Between January 2010 and December 2017, 998 individuals with diagnosed LC (squamous cell carcinoma) were found. Eligible patients were identified in a 3-stage exclusion process as summarized in Fig. 1. First, patients meeting the age criterion were selected; second, patients with follow-up shorter than 3 years were excluded; finally, patients with smoking history shorter than 30 pack-years or having pre-existing lung cancer were removed.

A detailed review of available medical history for each individual meeting inclusion criteria was performed. In Johns Hopkins Hospital, the electronic medical record (EMR) software storing patients’ medical history is called EPIC^®^ (Verona, Wisconsin, United States). The study protocol involved for each enrolled patient: screening in EPIC all chest CT exams and other clinical notes to identify also pulmonary nodules detected outside Johns Hopkins Hospital or documented by outside providers. An additional EMR feature allows access to data of the largest outside imaging providers in Maryland, USA (American Radiology and Advanced Radiology). Their database was also searched for any CT exams that might have not been visible in EPIC. For every CT found, we analyzed radiological reports in search for any detected pulmonary nodule larger than 4 mm. Size, number, change in size and location of detected nodules was extracted from radiological reports. Clinical and demographic characteristics such as sex, age, race, smoking pack-years, TNM-staging, tumor site were recorded.

### Statistics

GraphPad Prism software (version 6.0, GraphPad Software, La Jolla, CA) was used to perform statistical analyses. The significance level for all calculations was p < 0.05.

## Results

### Patient characteristics

Following identification and exclusion protocols (Fig. 1), 151 of 998 laryngeal cancer patients meeting inclusion criteria were analyzed. Demographic features of this population are found in Table 1. The majority of the group were men (75.5%) and included patients with early, locally advanced, and advanced LC, fairly evenly divided between glottic (38.4%) and supraglottic (45.0%) cancer.

**Table 1:** Patient demographics, demographics of patients in the NLST study (N = 53 454)^9^ and laryngeal cancer staging (N = 151).

		Johns Hopkins Hospital Cohort		National Lung Screening Trial^9^		Chi-square test (p-value)
		Patients (n)	Percentage^a^	Patients (n)	Percentage	
Gender	Male	114	75.50%	31 532	58.90%	<0.0001
	Female	37	24.50%	21 922	41.10%	
Age (yrs)	Mean ± SD	70 ± 6.9	—	—	—	
Race/ethnicity	White	100	66.20%	48 549	91.00%	<0.0001
	Black	44	29.10%	2376	4.40%	
	Other	6	4.00%	2157	4.00%	
Smoking history (pack yrs)	30–49	91	60.30%	—	—	
	50–99	50	33.10%	—	—	
	≥100	10	6.60%	—	—	
T staging (TNM^b^)	T1	42	27.80%	—	—	
	T2	38	25.20%	—	—	
	T3	38	25.20%	—	—	
	T4	20	13.30%	—	—	
N staging (TNM^b^)	N0	99	65.60%	—	—	
	N1	8	5.30%	—	—	
	N2	26	17.20%	-	—	
Tumor location	Glottic	58	38.40%	—	—	
	Supraglottic	68	45.00%	—	—	
	Subglottic	4	2.60%	—	—	

^a^Totals may not equal 151, as data was not available for all patients.

^b^7^th^ Edition of the AJCC TNM Classification (2010).

### Pulmonary nodules in laryngeal cancer patients

During the study period, a total of 746 patient-years were analyzed and 514 CT reports found. In seventy-eight patients (52%) a total of 219 PN were reported (Table 2). The majority of patients (52.5%) with positive findings had small pulmonary nodules with a maximum size of 4 to 5 mm, but the remainder had nodules significantly larger. Most frequently patients had 3 or more nodules detected over the screened period (48.7%), whereas only one nodule was found in 24 out of 78 patients (30.8%). The most common locations for PN were in the superior and inferior lobes (both 34.9%). In follow-up chest imaging, in most cases the detected nodules did not change in size (65.4%). Nevertheless, in 17 patients (21.8%) lung nodules increased in size on repeat imaging.

**Table 2:** Characteristics of detected pulmonary nodules.

Characteristics of detected pulmonary nodules	N (%)
**Size of the biggest nodule (mm)**	
4–5-Apr	41 (52.5)
6–8-Jun	18 (23.1)
9+	19 (24.4)
**Number of detected nodules in one patient**	
1	24 (30.8)
2	16 (20.5)
3+	38 (48.7)
**Size change in follow-up CTs**	
No change	51 (65.4)
Increase	17 (21.8)
Decrease	4 (5.1)
Resolved	6 (7.7)
**Location**	
Superior lobe	37 (34.9)
Middle lobe	16 (15.1)
Inferior lobe	37 (34.9)
Subpleural	16 (15.1)

**Figure 2:**
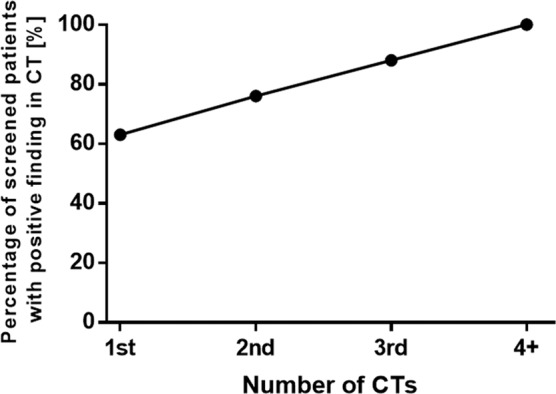
Percentage of patients with detected pulmonary nodules by number of CT scans. Only 63% pulmonary nodules were detected in the first year of CT imaging performed, and the rate of detected pulmonary nodules increased over the years as the number of CT scans increased.

**Figure 3:**
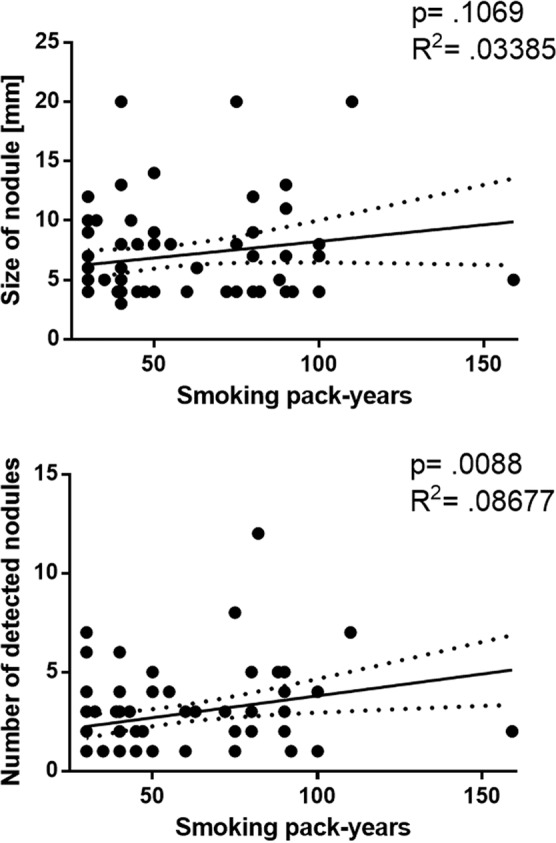
Correlation between size of detected pulmonary nodules, number of detected nodules and smoking history in pack-years.

In the analysed cohort, white race and smoking history over 40 pack-years were associated with greater risk of pulmonary nodule detection (p = 0.037 and p = 0.044, respectively). Sex, age, tumour site, T and N status were not significantly associated (Table 3). Because the design of the National Lung Screening Trial involved serial imaging over the course of three years, we were interested in how findings were detected in our cohort over the course of the three year inclusion criteria. In 49 cases (63%) pulmonary nodules were detected in the first CT imaging performed. Ten additional cases (13%) were identified by 2^nd^ CT, and ten more cases (13%) by the 3^rd^ CT. In 9 patients (11%), pulmonary nodules were detected in 4th or following chest CT (Fig. 2). Therefore, it appears that serial CT scan does improve the detection of pulmonary nodules over time, similar to findings in the National Lung Screening Trial^9^. Finally, we sought to identify factors associated with high-risk features of pulmonary nodules (Fig. 3)^11,12^. There was no correlation between the size of the nodule and smoking history in pack-years (p = 0.107, R^2^ = 0.03385). However, there was a weak correlation between the number of detected nodules and smoking history in pack years (p = 0.0088, R^2^ = 0.08677, respectively).

**Table 3:** Demographic and tumor characteristics stratified by presence of pulmonary nodule.

Variables	Pulmonary Nodules		*P* value
	No (%)	Yes (%)	
**Sex**			
Male	58 (50.9)	56 (49.1)	0.274^a^
Female	15 (40.5)	22 (59.5)	
**Race**			
White	42 (42.0)	58 (58.0)	0.037^a^
Non-white	30 (60.0)	20 (40.0)	
**Age (years)**			
Under 70	35 (47.9)	38 (52.1)	0.924^a^
Over 70	38 (48.7)	40 (51.3)	
**Smoking history (years)**			
Under 40	30 (60.0)	20 (40.0)	0.044^a^
Over 40	43 (42.6)	58 (57.4)	
**Tumor site**			
Glottic	27 (46.6)	31 (53.4)	0.961^a^
Supraglottic	32 (47.1)	36 (52.9)	
Transglottic	4 (57.1)	3 (42.9)	
Subglottic	2 (50.0)	2 (50.0)	
**T-staging**			
T1–T2	40 (50.0)	40 (50.0)	0.316^a^
T3–T4	24 (41.4)	34 (58.6)	
**N-status**			
N0	47 (47.5)	52 (51.5)	0.549^a^
N+	15 (41.7)	21 (58.3)	

^a^Pearson Chi-squere test.

### Secondary lung cancer in laryngeal cancer patients

In the analysed group, SLC was diagnosed in 9 (6%) patients during the course of the study. Affected patients were predominantly men (78%) with mean age of 76 (range from 70 to 84) and mean smoking history of 72 pack-years (range from 30 to 150 pack years). There was an average of five years between the primary diagnosis of LC and diagnosis of SLC (range from 1 to 12). Six out of nine (67%) patients who developed SLC were primarily diagnosed with supraglottic laryngeal cancer. The remaining 3 cases (33%) had a primary diagnosis of glottic cancer. Demographic and clinical data are presented in Table 4.

**Table 4:** Demographic and tumour associated characteristics of patients diagnosed with secondary lung cancer (SLC).

Pt ID	Age	Race	Sex	LC site	LC T-status	LC N-status	Smoking pack-years	LC diagnosis year	SLC diagnosis year	Years to SLC
1	84	w	M	Supraglottic	T1	N0	56	2010	2017	7
2	77	w	M	Supraglottic	T2	N0	100	2008	2012	4
3	71	o	M	Glottic	T2	N0	30	2003	2015	12
4	76	w	M	Glottic	T2	N0	150	2007	2015	8
5	83	w	M	Supraglottic	T3	N0	50	2012	2015	3
6	70	b	F	Supraglottic	T4	N0	50	2012	2014	2
7	72	w	F	Supraglottic	T2	N1	60	2005	2013	8
8	72	w	M	Supraglottic	T3	N2	45	2013	2014	1
9	79	w	M	Glottic	T4	Ni	110	2010	2012	2

In the analysed cohort, age over 70 years was the only factor significantly associated with greater risk of secondary lung cancer development (p = 0.003) (Table 5). Sex, race, smoking history, tumour site, T and N status were not significantly associated (*p* values: 1.00, 0.718, 0.272, 0.772, 1.00, 1.00, respectively).

**Table 5:** Demographic and tumor characteristics stratified by presence of secondary lung cancer.

Variables	Lung cancer		*P* value
	No (%)	Yes (%)	
**Sex**			
Male	107 (93.9)	7 (6.1)	1.00^a^
Female	35 (94.6)	2 (5.4)	
**Race**			
White	93 (93.0)	7 (7.0)	0.718^a^
Non-white	48 (96.0)	2 (4.0)	
**Age (years)**			
Under 70	73 (100.0)	0 (0.0)	0.003^a^
Over 70	69 (88.5)	9 (11.5)	
**Smoking history (years)**			
Under 40	49 (98.0)	1 (2.0)	0.272^a^
Over 40	93 (92.1)	8 (7.9)	
**Tumor site**			
Glottic	55 (94.8)	3 (5.2)	0.772^a^
Supraglottic	62 (91.2)	6 (8.8)	
Transglottic	7 (100.0)	0 (0.0)	
Subglottic	4 (100.0)	0 (0.0)	
**T-staging**			
T1–T2	75 (93.8)	5 (6.2)	1.00^a^
T3–T4	54 (93.1)	4 (6.9)	
**N-status**			
N0	93 (93.9)	6 (6.1)	1.00^a^
N+	34 (94.4)	2 (5.6)	

^a^Fisher’s Exact Test.

## Discussion

Evidence-based medicine has established that a discrete group of high-risk patients benefits from annual lung cancer CT screening. The management of detected pulmonary nodules, appropriate risk stratification and follow-up strategies in this cohort is an intensive area of investigation in pulmonology, oncology, public health, radiology and many others after the introduction of USPSTF guidelines^8^. However, even though the results of the biggest clinical trial justifying the introduction of annual chest screening in smokers was published over 5 years ago, still little is known about the frequency of radiological findings or clinical benefits of the screening program in a large population of potential patients – those with previously diagnosed malignancy, who were excluded from the aforementioned study.

Knowing that smoking is a causative agent in many types of cancer, it seems justifiable to include those patients in annual screening problems as they often meet official USPTF criteria. In particular, it is widely known that head and neck cancer (HNSCC) patients are at significant risk of developing secondary lung malignancy. It has been reported that 5–19% of primary HNSCC patients develop SLC^3–6^ and this number is substantially higher than for smokers in general. The National Lung Screening Trial^9^ included 53 454 smokers meeting 55–74 age criterion and 30 pack-years smoking history or having quitted within past 15 years. Treatment for, or evidence of, any cancer other than nonmelanoma skin cancer or carcinoma *in situ* in the 5 years prior to eligibility assessment was one of the major exclusion criterion. Pulmonary nodules were detected in 24.2% and malignant transformation observed in 4% of this studied population. By comparison, in our cohort we report a 52% rate of pulmonary nodules and a 6% rate of secondary lung cancer, rates that would seem to obviously justify annual screening when compared to the rates that merit annual CT screening in those without a prior diagnosis of malignancy. We acknowledge that it is possible that to some extent the rate of pulmonary nodules in our cohort depends on the differences in demographics between our study and NLST. Our cohort has a higher representation of non-white ethnicity patients (29.1% vs. 4.4% in NLST, Table 1.), who proved to be significantly less frequently diagnosed with pulmonary nodules (Table 3).

Our results are consistent with the previous report of Green *et al*.^7^, who reported that in a cohort of 400 HNSCC patients pulmonary nodules were present in 58% of patients and the lung malignancy rate equalled 6%. However, the aforementioned study included patients regardless of their age or smoking history and a considerable percentage of included patients had oropharyngeal cancer, where smoking is not as pronounced a risk factor as in laryngeal cancer. Therefore we believe that our study is unique and fills gap in the literature regarding prevalence of pulmonary nodules and secondary lung cancer in a carefully selected group of laryngeal cancer patients meeting USPSTF criteria for annual chest screening. The reported prevalence of PN and SLC in our study is higher than in the National Lung Screening Trial and surprisingly, white race proved to be significantly associated with greater risk of pulmonary nodule presence. To our knowledge, this finding has not been previously published in the available literature. The other factor positively associated with presence of PN, namely smoking history over 40 pack-years was expected and previously proved^13,14^.

Lung cancer is the most common secondary malignancy in head and neck patients^15,16^. The reported prevalence of secondary primary lung cancer in head and neck cancer patients ranges from 5–19%^3–6^. In our study, the prevalence of secondary lung malignancy (6%) was quite low compared to the literature. However, unlike other studies we excluded all patients who were diagnosed with lung cancer within first 12 months following LC diagnosis and we had a homogenous population regarding primary tumour site. We show that the time between diagnosis of primary head and neck malignancy and secondary lung cancer varies between cases. The average of five years between diagnoses indicates that a substantial number of patients may develop lung cancer after their completed 5-year surveillance for laryngeal cancer, further evidence for the importance of continued screening for those patients that meet USPSTF criteria.

We admit there are some limitations to our study, the first being its retrospective character. Secondly, approximately 50% of patients meeting the age criterion were excluded based on the poor quality of follow-up record. Finally, another factor limiting our results is unreliable documentation of smoking history. To select patients definitely meeting USPSTF criteria for annual lung screening we were forced to exclude 172 patients based on their declared smoking history. In majority of cases, pack-years were not recorded in any form in their medical chart. As smoking history is one of the most important criterion in the introduced guidelines, accurate reporting of tobacco exposure in the standardized measure of pack-years should be a necessity in every day practice.

## Conclusion

The incidence of PN and SLC in patients with LC is high. Many patients with laryngeal cancer meet the formal guidelines for USPSTF screening, and should be screened annually according to evidence-based medicine for the early detection of secondary lung cancers.

## References

[1] Surveillance, Epidemiology, and End Results (SEER) Program (www.seer.cancer.gov) SEER*Stat Database: Incidence - SEER 9 Regs Research Data, Nov 2017 (1973–2015) Katrina/Rita Population Adjustment - Linked To County Attributes - Total U.S., 1969–2016 Counties, National Cancer Institute, DCCPS, Surveillance Research Program, released April 2018, based on the November 2017 submission.

[2] Lewis A, Kang R, Levine A, Maghami E (2015). The New Face of Head and Neck Cancer: The HPV Epidemic. Oncology (Williston Park)..

[3] Dikshit RP (2005). Risk factors for the development of second primary tumors among men after laryngeal and hypopharyngeal carcinoma. Cancer.

[4] Sjögren EV, Snijder S, van Beekum J, Baatenburg de Jong RJ (2006). Second malignant neoplasia in early (TIS-T1) glottic carcinoma. Head Neck.

[5] Farhadieh RD, Salardini A, Yang JL, Russell P, Smee R (2010). Diagnosis of second head and neck tumors in primary laryngeal SCC is an indicator of overall survival and not associated with poorer overall survival: A single centre study in 987 patients. J. Surg. Oncol..

[6] Hsu Y-B (2008). Second Primary Malignancies in Squamous Cell Carcinomas of the Tongue and Larynx: An Analysis of Incidence, Pattern, and Outcome. J. Chinese Med. Assoc..

[7] Green R (2017). Analysis of the incidence and factors predictive of outcome in patients with head and neck cancer with pulmonary nodules. Head Neck.

[8] Humphrey, L. *et al*. Screening for Lung Cancer: Systematic Review to Update the U.S. Preventive Services Task Force Recommendation. (Agency for Healthcare Research and Quality (US), 2013). 24027793

[9] National Lung Screening Trial Research Team *et al*. Reduced Lung-Cancer Mortality with Low-Dose Computed Tomographic Screening. *N. Engl. J. Med*. **365**, 395–409 (2011). 10.1056/NEJMoa1102873 PMC4356534 21714641

[10] Piersiala K, Akst LM, Hillel AT, Best SR (2019). Clinical practice patterns in laryngeal cancer and introduction of CT lung screening. Am. J. Otolaryngol. - Head Neck Med. Surg..

[11] Larici AR (2017). Lung nodules: size still matters. Eur. Respir. Rev..

[12] MacMahon H (2017). Guidelines for Management of Incidental Pulmonary Nodules Detected on CT Images: From the Fleischner Society 2017. Radiology.

[13] He Y-T (2018). Risk factors for pulmonary nodules in north China: A prospective cohort study. Lung Cancer.

[14] Wong ML (2018). Age, comorbidity, life expectancy, and pulmonary nodule follow-up in older veterans. PLoS One.

[15] Jones AS (1995). Second primary tumors in patients with head and neck squamous cell carcinoma. Cancer.

[16] Herranz González-Botas J, Varela Vázquez P, Vázquez Barro C (2016). Segundos tumores primarios en cáncer de cabeza y cuello. Acta Otorrinolaringológica Española.

